# Resolution of Thyroid Acropachy in a Patient Treated With Teprotumumab: A Case Report and Review of Mechanisms

**DOI:** 10.1155/crie/5544869

**Published:** 2025-07-18

**Authors:** Soumya Chatterjee

**Affiliations:** Cleveland Clinic Lerner College of Medicine of Case Western Reserve University, Department of Rheumatic and Immunologic Diseases, Medical Specialty Institute, Cleveland Clinic, Cleveland, Ohio, USA

## Abstract

Graves' disease is an autoimmune thyroidopathy associated with hyperthyroidism and nonendocrine manifestations such as thyroid eye disease (TED), pretibial myxedema, and thyroid acropachy. Thyroid acropachy is an uncommon but debilitating condition, typically characterized by digital clubbing, soft tissue swelling, and periosteal new bone formation in the hands and feet. This condition often accompanies TED and dermopathy, but effective treatments remain elusive. The first documented case of thyroid acropachy successfully treated with teprotumumab, a monoclonal antibody targeting the insulin-like growth factor-1 receptor (IGF-1R), is reported here. A 49-year-old female with a history of Graves' disease developed severe musculoskeletal symptoms, including clubbing and periosteal new bone formation. Despite initial therapies with rituximab and intravenous immunoglobulin showing limited benefit, treatment with teprotumumab, primarily prescribed for TED, led to significant clinical and radiological improvement. After completing eight cycles of teprotumumab, the patient's musculoskeletal pain resolved, clubbing regressed, and radiologic findings of periosteal bone formation diminished. This case highlights the potential of teprotumumab as a novel therapeutic option for thyroid acropachy and suggests that IGF-1R plays a crucial role in its pathogenesis. While this report presents promising results, further studies are needed to confirm the efficacy of teprotumumab in treating thyroid acropachy and better understand its long-term effects on this rare condition.

## 1. Introduction

Robert James Graves, [1] an Irish physician, first described Graves' disease in 1835. It is an autoimmune thyroidopathy characterized by goitrous hyperthyroidism and associated nonendocrine manifestations such as thyroid eye disease (TED), pretibial myxedema, and thyroid acropachy [2]. It affects approximately 1.2% of the global population. Women are about 10 times more likely to develop it than men. Between 20 and 40 new cases are reported annually per 100,000 individuals, and the lifetime risk is around 3% for women and 0.5% for men. It typically presents between the ages of 30–50 [2].

### 1.1. Clinical and Laboratory Manifestations

#### 1.1.1. Goitrous Hyperthyroidism

Most patients develop a symmetrically enlarged thyroid associated with hyperthyroidism [2]. The thyroid-stimulating hormone (thyrotropin) receptor (TSHR) is a key autoantigen in this process, as thyrotropin receptor autoantibodies (TRAbs) stimulate thyroid growth and excessive thyroid hormone production. Symptoms include staring, lid lag, heat intolerance, hyperhidrosis, tremors, palpitations, anxiety, weight loss, oligomenorrhea, and shortness of breath. Atrial fibrillation and heart failure occur in some cases, especially in older adults. Osteopenia or osteoporosis is common [2]. Thyroid histology typically shows follicular hyperplasia and lymphocytic infiltration.

### 1.2. Extrathyroidal Manifestations

#### 1.2.1. TED

TED is unique to Graves' disease [2] and is seen in 25% of patients [3]. The TSHR, along with the insulin-like growth factor-1 receptor (IGF-1R), activates orbital inflammation, resulting in proptosis (exophthalmos), diplopia, and periorbital swelling. Severe cases may result in optic neuropathy and blindness. Smoking, older age (over 50), and male sex are risk factors for more severe presentations.

#### 1.2.2. Pretibial Myxedema

This condition involves the skin, usually over the shins, and affects 0.5%–4.3% of patients. Symptoms include hyperpigmented, raised, and scaly lesions with a peau d'orange texture due to glycosaminoglycan accumulation in the dermis. Fibroblast stimulation via IGF-1R plays a key role in this condition, often seen in patients with severe orbitopathy and elevated TRAb levels [4].

#### 1.2.3. Thyroid Acropachy

It is a rare manifestation of Graves' disease that causes soft tissue swelling of the hands and feet, digital clubbing, and radiologic findings of smooth-flowing periosteal reaction with new bone formation at the metacarpals and phalanges [3, 4]. It is almost always associated with thyroid dermopathy and TED and usually becomes apparent after the diagnosis and treatment of Graves' hyperthyroidism. As with dermopathy, tobacco use is associated with acropachy in 75% of men and 81% of women [5].

The most common presentation of thyroid acropachy is clubbing of the fingers and toes. It resembles other causes of clubbing and affects 20% of thyroid dermopathy patients [4]. As the condition progresses, the fingers and toes develop symmetrical fusiform swellings, which can be disfiguring [5]. Sometimes, a single digit can also be involved [4]. The complete clinical picture consists of swelling of the fingers and toes associated with a periosteal reaction of the underlying bones. TED and Graves' dermopathy almost always accompany acropachy. Unlike hypertrophic osteoarthropathy, there is no involvement of the joints or localized hyperemia. About 50% of patients with acropachy have a severe form of dermopathy, including elephantiasis. Some patients are affected by painful periostitis and are unable to use their digits due to extreme pain and swelling, requiring anti-inflammatory agents and even narcotic analgesics [5].

Radiographic findings include fusiform soft tissue swelling of the fingers and toes and symmetrical subperiosteal bone formation affecting the metacarpals, metatarsals, and phalanges [4]. Unlike hypertrophic osteoarthropathy, the subperiosteal reaction does not always involve the long bones of the forearms and the legs [5]. Focal radionuclide accumulation in affected areas may be detected by 99mTc-MDP bone scintigraphy.

Histologic features in thyroid acropachy are similar to those of thyroid dermopathy. So far, only one histological study has been published that showed nodular periosteal fibrosis with subperiosteal bone formation and fibrosis [6]. The process is believed to be initiated by immune-mediated activation of periosteal fibroblasts and mucin deposition [3].

Despite the lack of specific studies, it is plausible that the pathogenesis of thyroid acropachy is similar to TED and dermopathy.

### 1.3. Treatment of Graves' Disease

#### 1.3.1. Hyperthyroidism

Management options for hyperthyroidism in Graves' disease include beta-blockers, antithyroid medications (thionamides), radioactive iodine, and thyroidectomy [2]. Thyroid storm, a life-threatening complication of hyperthyroidism, may require glucocorticoids and iodine elixirs.

### 1.4. Nonendocrine Manifestations

#### 1.4.1. TED

Treatment involves both nonimmunosuppressive and immunosuppressive therapies. Selenium supplements and statins may help reduce orbitopathy incidence, while intravenous glucocorticoids remain the first-line therapy [7]. Mycophenolate mofetil, in combination with glucocorticoids, has shown superior efficacy compared to glucocorticoid monotherapy. Other agents, such as methotrexate, azathioprine, cyclosporine, rituximab, and tocilizumab, have also demonstrated efficacy in some cases [7].

##### 1.4.1.1. Surgical and Radiation Therapy

Orbital decompression surgery and intensity-modulated orbital radiation therapy are considered for severe cases, especially when sight-threatening complications arise [7].

##### 1.4.1.2. Teprotumumab

This FDA-approved monoclonal antibody, which targets IGF-1R, has shown promise in treating TED by reducing inflammation and disease progression. It is administered via intravenous infusion over a series of treatments [7, 8].

#### 1.4.2. Pretibial Myxedema

Topical and intralesional glucocorticoids are the mainstays of treatment for mild cases. Pentoxifylline and rituximab have also been used with varying success. Teprotumumab has been reported to help in some cases of pretibial myxedema, though it is not FDA-approved for this indication [9].

#### 1.4.3. Thyroid Acropachy

Though the pathomechanism of the nonendocrine manifestations of Graves' disease (TED, pretibial myxedema, and thyroid acropachy) seems to be similar [10], and the TSHR and IGF-1 receptor interaction likely plays a central role in their development, it has been unclear if teprotumumab is effective in treating thyroid acropachy [9]. Therefore, no clear evidence or clinical guidelines currently support its use in this condition. A case of symptomatic thyroid acropachy is presented here, where, for the first time, teprotumumab was shown to successfully alleviate the musculoskeletal symptoms and revert the associated radiologic findings.

## 2. Case Presentation

**Figure 1:**
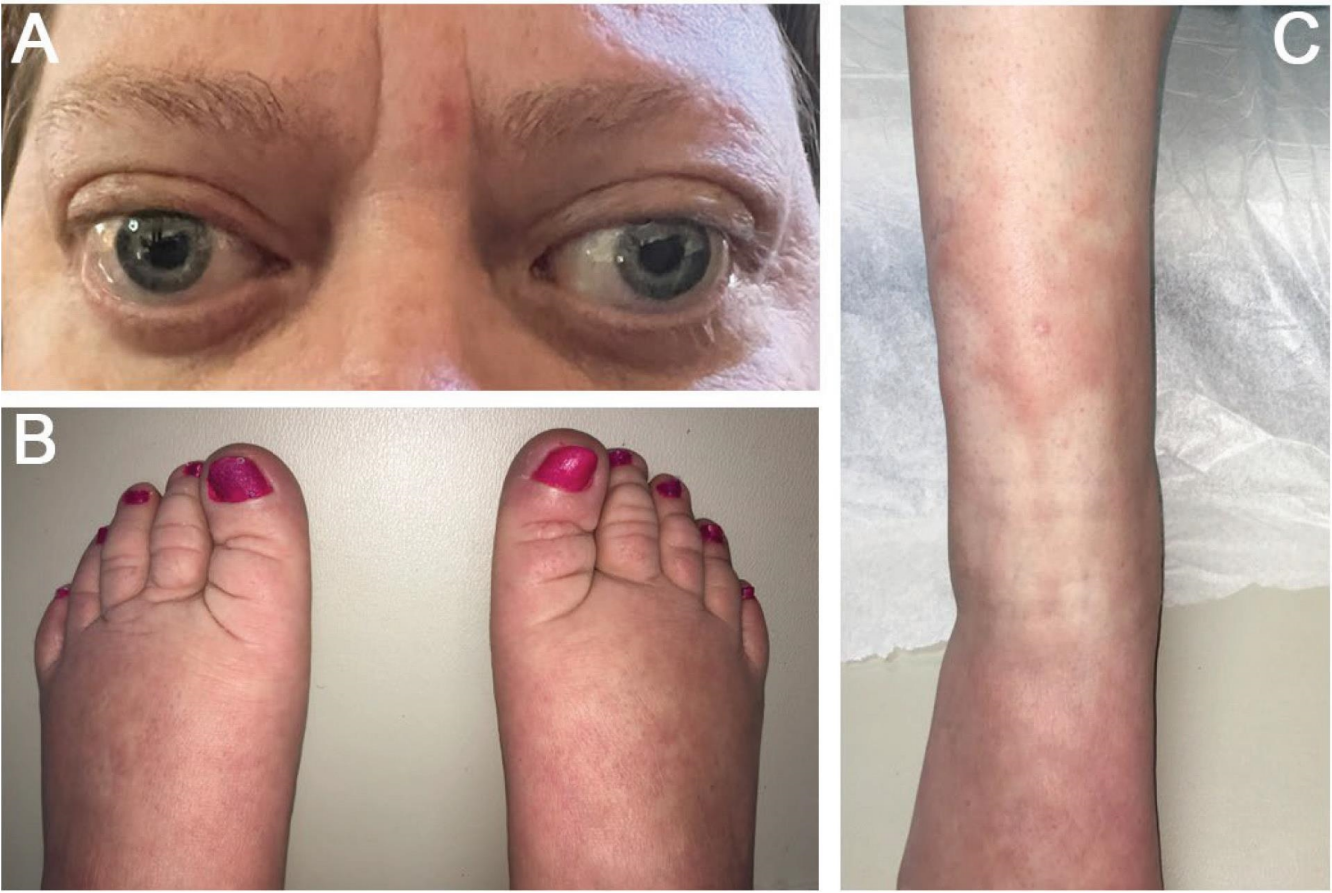
A 49-year-old female with Graves' disease showing (A) exophthalmos, (B) swollen toes with multiple dorsal transverse creases (Graves' dermopathy), and (C) circumscribed thickening of the skin on her right shin (Graves' dermopathy).

A 49-year-old female was first seen in the rheumatology clinic 6 years ago. About 8 years ago, she developed tachycardia, syncopal episodes, heat intolerance, and progressive exophthalmos (Figure 1A). She was diagnosed with Graves' disease by her endocrinologist, for which she had a subtotal thyroidectomy a month after the onset of symptoms, leading to postsurgical hypothyroidism, requiring levothyroxine 137*µ*g daily. Though she became euthyroid, she continued to experience fatigue. She also needed multiple eye surgeries, including bilateral orbital decompression, to improve her exophthalmos, with unsatisfactory results. Two to 3 months later, she noticed skin redness and induration on her shins that progressed (pretibial myxedema) (Figure 1C).

When she was first seen at the rheumatology clinic, her main symptoms were bilateral foot pain and tingling. She also complained of stiffness and puffiness in her fingers and circumscribed thickening of the skin on her shins (Figure 1C). There was pain and stiffness in her toes and the proximal interphalangeal joints of her fingers. She had skin thickness and swelling in her fingers, lower legs, feet, and toes, which started a year ago. She had been unable to fit into her stiletto boots for work since this started. In addition, she complained of deep-seated pain in her bilateral thighs.

**Figure 2:**
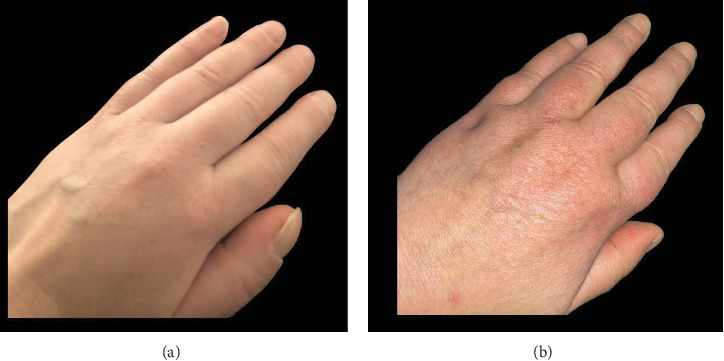
Regression of thyroid acropachy with teprotumumab: (A) puffy fingers and finger clubbing (thyroid acropachy), (B) Regression of clubbing and soft tissue swelling of fingers following eight three-weekly infusions of teprotumumab.

On physical examination, her vital signs were normal. She had bilateral exophthalmos (Figure 1A), puffy fingers and bilateral finger clubbing (Figure 2A), swollen toes with multiple dorsal transverse creases (Figure 1B), and patches of erythematous indurated skin on the shins (pretibial myxedema) (Figure 1C). There was tenderness of the metacarpophalangeal and proximal interphalangeal joints, wrists, ankles, and toes.

### 2.1. Investigations

Her initial laboratory results are shown in Table 1.

**Figure 3:**
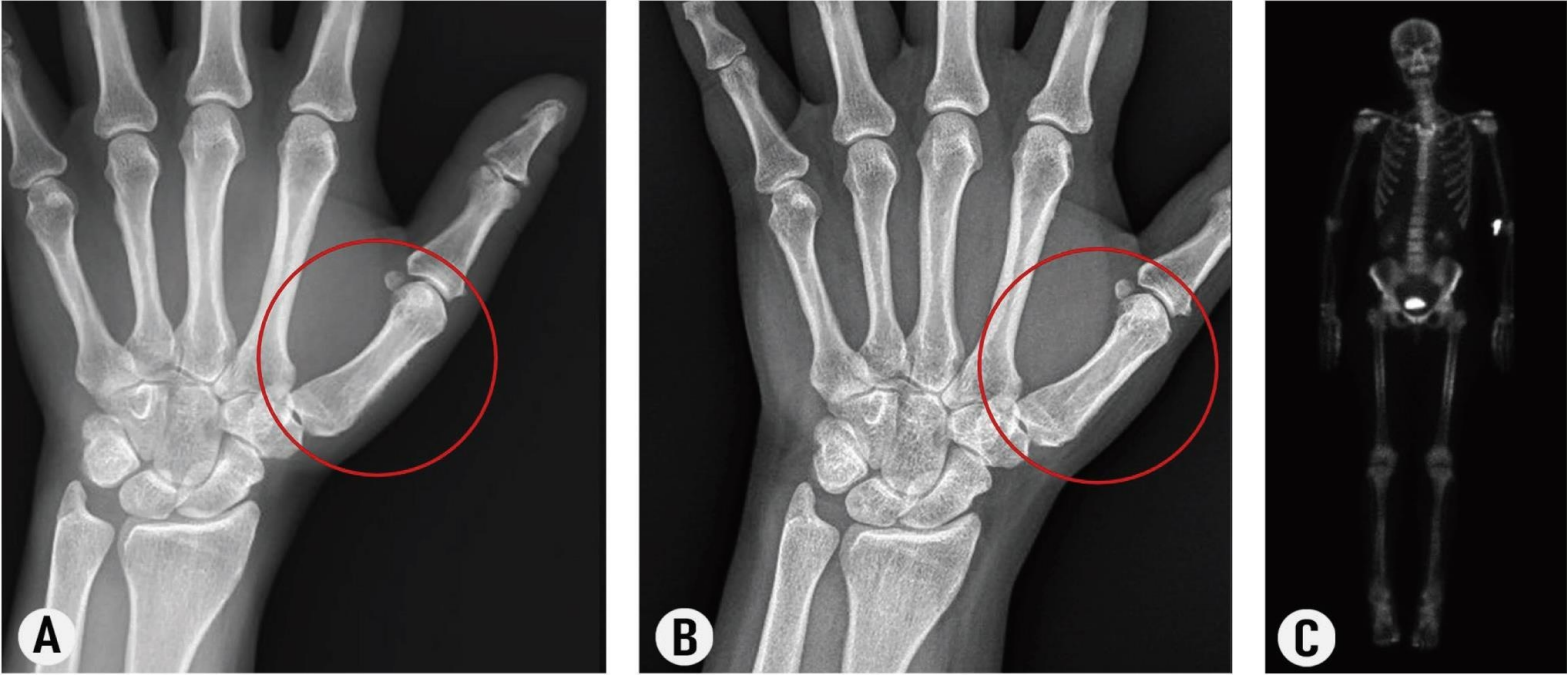
Regression of radiographic and bone scintigraphic findings of thyroid acropachy with teprotumumab: (A) left-hand radiograph showing evidence of periosteal reaction along the radial cortex of the first metacarpal, consistent with thyroid acropachy, (B) left-hand radiograph showing regression of the periosteal reaction following eight 3-weekly infusions of teprotumumab, and (C) 99mTc MDP bone scintigraphy showing bilateral periosteal activities within the midshafts of the femurs, proximal tibiae, and first metacarpals (periostitis).

A CT scan of the orbits showed diffusely enlarged extraocular muscles and bilateral proptosis, consistent with TED. Hand radiographs showed evidence of a periosteal reaction along the radial cortices of the first metacarpals, consistent with thyroid acropachy (Figure 3A). To demonstrate the cause of her bilateral thigh pain, she underwent 99mTc MDP bone scintigraphy with 3-h delayed images that showed periosteal activity within the midshafts of the femurs and proximal tibiae, representing periostitis (Figure 3C) similar to cases reported in the literature [4, 5]. Mild activity was also noted bilaterally within the first metacarpals (Figure 3C).

Based on the available evidence, she was diagnosed with thyroid dermopathy (Figure 1B,C) and acropachy (Figures 1B, 2A, and 3A).

### 2.2. Treatment

As her TSH level was suppressed (Table 1) on her current dose of levothyroxine, her dose was reduced to 125 µg 5 days a week. Based on the EUGOGO guidelines, she was also started on pentoxifylline 400 mg thrice daily and selenium 200 µg daily [7]. For her tachycardia, she was on metoprolol 100 mg twice daily. For diffuse pain, she was started on pregabalin 150 mg three times daily and duloxetine 30 mg twice daily. For pretibial myxedema, triamcinolone acetonide, 0.1% topical cream, twice daily, was prescribed.

In addition, she was initially treated with intravenous rituximab infusions for four cycles (8 infusions) [2, 11]. As the benefit was questionable, monthly intravenous immunoglobulin infusions (2 g/Kg/cycle) [11] were tried for 6 months, with minimal benefit.

Then, as her TED progressed, she was started on teprotumumab-trbw intravenous infusions [8]. She completed eight cycles (10 mg/kg for the first infusion, followed by 20 mg/kg every 3 weeks for seven additional infusions) [8]. After completing her teprotumumab course, she noticed a significant improvement in her TED symptoms and the pain in her hands, thighs, and feet. Furthermore, her clubbing and soft tissue swelling of fingers (Figure 2B) and feet reverted. Also, the radiologic changes of periosteal new bone formation affecting the first metacarpals regressed (Figure 3B).

#### 2.2.1. Patient's Narrative

*“Before starting teprotumumab treatment*, *life was challenging. The severe swelling in my feet from thyroid acropachy meant I could only wear slip-on shoes. The condition also caused intense pain in my bones from new bone growth*, *affecting both my feet and hands*.

*However*, *after just one cycle of teprotumumab*, *I began to notice a reduction in the swelling in both my feet and hands*, *and the pain started to improve with each subsequent treatment. The swelling continued to decrease throughout eight cycles*, *and the pain and swelling in my hands and fingers were entirely resolved. While teprotumumab was initially prescribed for my thyroid eye disease*, *it also significantly alleviated my acropachy symptoms.”*

## 3. Discussion

This case demonstrates for the first time that, in addition to TED [8, 12] and pretibial myxedema [13, 14], symptomatic thyroid acropachy can be successfully treated with teprotumumab, alleviating the clubbing, musculoskeletal symptoms, and radiologic findings associated with this debilitating condition.

The implications of these findings are profound. Even though thyroid acropachy is rare, it can be quite a debilitating condition that has not been successfully treated with disease-modifying therapies to date. The literature discusses available treatments for TED and dermopathy, including conventional disease-modifying antirheumatic drugs and biologic therapies such as rituximab, tocilizumab, and intravenous immunoglobulin [2, 3, 7]. However, the results are inconsistent and not always positive. In the present case, both rituximab and intravenous immunoglobulin infusions were ineffective in alleviating the nonendocrine (autoimmune) manifestations of Graves' disease.

Although the pathogenesis of thyroid acropachy remains incompletely understood, it is presumed to involve the same autoimmune mechanisms underlying the other extrathyroidal manifestations of Graves' disease, such as TED and pretibial myxedema. Therefore, even though the role of IGF-1R in the development of thyroid acropachy remains uncertain, the severity of the patient's symptoms warranted a therapeutic trial of teprotumumab, as it made pathophysiological sense and was already being used for the treatment of coexisting TED. In this context, its effectiveness is supported by its proposed mechanism of action and the presumed shared pathogenesis between TED, pretibial myxedema, and thyroid acropachy. While the only histologic study of thyroid acropachy, published in 1959, did not include cellular characterization [6], based on similarities to TED [15], it is reasonable to hypothesize that in thyroid acropachy, bone marrow-derived fibrocytes migrate and infiltrate the periosteal tissues of the metacarpals, metatarsals, and phalanges. Upon recruitment to these sites of tissue injury, they differentiate into fibroblasts, which, under autoimmune stimulation, likely increase the production and deposition of mucopolysaccharides (glycosaminoglycans) within the soft tissues and periosteum [4, 5, 15]. By inhibiting IGF-1R activation, Teprotumumab interferes with the physical and functional interaction of IGF-1R and TSHR, as well as their synergistic signaling in fibrocytes and fibroblasts. This inhibition interferes with TSH-driven proinflammatory and profibrotic signaling pathways, such as the downstream activation of PI3-kinase/Akt and NF-κB, the release of cytokines like TNF-α, IL-6, IL-8, and B-cell activating factors, as well as the excessive production of glycosaminoglycans (especially hyaluronan), which contribute to tissue expansion and remodeling [16]. The central role of IGF-1 and its crosstalk with TSHR and TRAb provided a rationale for initiating a trial of teprotumumab in this patient. As anticipated, the outcome was favorable, supporting the underlying hypothesis.

Blocking IGF-1R has shown biological and clinical advantages beyond its use in TED, highlighting its wider potential as an antifibrotic and anti-inflammatory therapy [16]. IGF-1 inhibition is being studied as a potential treatment for other endocrine conditions, such as acromegaly and diabetes mellitus [16, 17]. Malignancies are the main conditions outside of TED, where IGF-1R inhibition has demonstrated therapeutic benefit. IGF-1R plays a role in the development and progression of various solid tumors, such as Ewing sarcoma, nonsmall cell lung cancer, and some types of breast cancer [18]. IGF-1R signaling influences the activation and trafficking of immune cells, indicating a broader role in fibroblast dysregulation associated with autoimmune diseases [19–21]. In experimental pulmonary fibrosis and scleroderma-related skin fibrosis models, IGF-1R inhibition reduces fibroblast proliferation and collagen deposition [22–25]. These findings offer a mechanistic basis for investigating teprotumumab as a potential therapy for thyroid acropachy, where IGF-1R inhibition could address pathological fibroblast activation, soft tissue expansion, and abnormal bone remodeling.

Though this case is worth reporting, it is essential to acknowledge that a single case study has inherent limitations, and its results should not be overinterpreted. We should not draw any definitive conclusions just yet, as the results of more extensive future studies might not be as convincing. In the present case, potential confounders could be delayed effects of prior therapies the patient had received, including carryover effects of rituximab and intravenous immunoglobulin infusions. Furthermore, we do not seem to know enough about the natural history of thyroid acropachy and whether, in some patients, it can spontaneously regress over time, as reported in the literature [5]. In a 40-patient study of thyroid acropachy, treated over 26 years, musculoskeletal pain subsided at the last office visit. Also, on the follow-up questionnaire, no patient complained of clubbing, and medical examiners rarely mentioned clubbing or acropachy during the final evaluation. However, the rate of clubbing resolution was unclear in this retrospective study. If the current patient's natural history of thyroid acropachy followed the same course reported in the above single-center study [5], teprotumumab received undue credit as the purported benefits could not be attributed to its use. Nevertheless, it should be worth noting that the current patient continued to experience ongoing and progressively worsening musculoskeletal pain, digital and leg/foot swelling, and clubbing for 8 years after the onset of Graves' disease, which only subsided after the teprotumumab course was completed, strongly suggesting a possible disease-modifying role of teprotumumab for thyroid acropachy.

Though teprotumumab has been proven to be an effective treatment for nonendocrine manifestations of Graves' disease and is FDA-approved for TED, it is a prohibitively expensive therapy [26]. A cost-effectiveness analysis used recommended dosing guidelines to estimate the costs of teprotumumab, rituximab, orbital radiotherapy, and intravenous methylprednisolone, standardized for a 70-kg patient with TED. Among these treatments, teprotumumab had the highest mean treatment-related cost at $386,424 ± $65,217 (standard deviation), followed by rituximab at $18,549 ± $2,556, orbital radiotherapy at $4316 ± $1183, and intravenous methylprednisolone at $4025 ± $1647. It was found that the treatment cost/change in “TED quality of life” (ΔGO-QoL) of teprotumumab was nearly 20 times that of rituximab and almost 50 times that of intravenous methylprednisolone [26].

Based on its proven efficacy in TED and dermopathy, this case study incentivizes further research into the safety and effectiveness of teprotumumab for thyroid acropachy, which can be resistant to the conventional therapies used for nonendocrine manifestations of Graves' disease. Additionally, cost-effectiveness studies should be conducted to assess the return on investment of this expensive treatment compared to biologic therapies such as rituximab, tocilizumab, and intravenous immunoglobulin. Moreover, teprotumumab has potential side effects, including infusion reactions, hyperglycemia, sensorineural hearing loss, and worsening of preexisting inflammatory bowel disease [2]. In addition, there have been reports of amyloid encephalopathy, which can have devastating consequences [2].

In conclusion, this case study supported the hypothesis that teprotumumab could effectively alleviate the signs and symptoms of thyroid acropachy, offering novel insights into the central role of IGF-1 in its pathogenesis. However, it should be emphasized that this is a hypothesis-generating case report, not definitive evidence of teprotumumab's effectiveness in reversing thyroid acropachy. This relatively safe but expensive biologic therapy can be considered for the treatment of symptomatic thyroid acropachy unresponsive to conventional therapies used for TED and dermopathy. However, though promising, the results should not change clinical practice yet. Further in-depth studies are necessary, including prospective clinical trials.

## Figures and Tables

**Table 1:** Initial laboratory results.

Laboratory tests	Normal value	Results
Complete blood count and differential count		
WBC (k/µL)	3.70–11.00	4.90
RBC (m/µL)	3.90–5.20	3.89
Hemoglobin (g/dL)	11.5–15.5	13.2
Hematocrit (%)	36.0–46.0	40.3
MCV (fL)	80.0–100.0	103.6
MCH (pG)	26.0–34.0	33.9
MCHC (g/dL)	30.5–36.0	32.8
RDW-CV (%)	11.5–15.0	13.1
Platelet count (k/µL)	150–400	351
MPV (fL)	9.0–12.7	9.5
Neutrophil (%)	—	41.3
Absolute neutrophil count (k/µL)	1.45–7.50	2.01
Lymph (%)	—	39.0
Absolute lymphocyte count (k/µL)	1.00–4.00	1.91
Mono (%)	—	11.4
Absolute monocyte count (k/µL)	<0.87	0.56
Eosin (%)	—	7.3
Absolute eosinophil count (k/µL)	<0.46	0.36
Baso (%)	—	1.0
Absolute basophil count (k/µL)	<0.11	0.05
Complete metabolic panel		
Protein, total (g/dL)	6.3–8.0	7.0
Albumin (g/dL)	3.9–4.9	4.2
Calcium (mg/dL)	8.5–10.2	8.3
Bilirubin, total (mg/dL)	0.2–1.3	0.2
Alkaline phosphatase (U/L)	32–117	52
AST (U/L)	13–35	13
Glucose (mg /dL)	74–99	90
BUN (mg /dL)	7–21	7
Creatinine (mg /dL)	0.58–0.96	0.82
Sodium (mmol/L)	136–144	139
Potassium (mmol/L)	3.7–5.1	4.2
Chloride (mmol/L)	97–105	105
CO_2_ (mmol/L)	22–30	23
Anion gap (mmol/L)	9–18	11
ALT (U/L)	7–38	<5
CK (U/L)	42–196	37 (L)
eGFR	>60	>60
Endocrine test results		
Thyroid-stimulating antibody (TSAb) (% normal)	<150	4100
TSH (µU/mL)	0.400–5.500	0.037
T3 (ng/dL)	79–165	108
Free T4	0.9–1.7	1.6
Thyroglobulin Ab (IU/mL)	<14.4	11.1
Thyroid peroxidase antibody (IU/mL)	<5.6	87.5
PTH, intact (pg/mL)	15–65	59
Urinalysis		
Color	Yellow	Yellow
Clarity	Clear	Cloudy
Glucose, urine	Negative	Negative
Bilirubin, urine	Negative	Negative
Ketones, urine	Negative	Negative
Specific gravity, Ur	1.005–1.030	1.009
Hemoglobin/blood, Ur	Negative	Negative
pH, urine	4.5–8.0	6.0
Protein, urine (mg/dL)	Negative	Negative
Urobilinogen	Normal	Normal
Nitrites	Negative	Negative
Leukocyte esterase	Negative	1+
WBC, urine (HPF)	0–5	0–5
RBC, urine (HPF)	0–3	0–3

## Data Availability

The data that support the findings of this study are available from the corresponding author upon reasonable request.
